# Study of *Artemisia ordosica* Krasch. Against Allergic Rhinitis Based on the P815 Mast Cell Degranulation Model, Network Pharmacology, Molecular Docking, and Molecular Dynamics

**DOI:** 10.3390/ijms27115122

**Published:** 2026-06-05

**Authors:** Mengmeng Wang, Jingming Zou, Qi Zhang, Xianxiang Bai, Si Wu, Yawei Hu, Xiaoyan Han, Na Han, Bin Xiao

**Affiliations:** 1 Development and Utilization Key Laboratory of Northeast Plant Materials, School of Traditional Chinese Materia Medica, Shenyang Pharmaceutical University, Shenyang 110016, China; 15037380861@163.com (M.W.); tnzq719@163.com (Q.Z.); 2 General Clinical Research Center, Ordos School of Clinical Medicine, Inner Mongolia Medical University, Ordos 017000, China; m15184699090@163.com (J.Z.); lucky_white@126.com (X.B.); wusijiayou@126.com (S.W.); weyeahu@163.com (Y.H.); 18648479785@163.com (X.H.); 3 Baotou Medical College, Inner Mongolia University of Science and Technology, Baotou 014040, China

**Keywords:** *Artemisia ordosica* Krasch., allergic rhinitis, mast cell degranulation, network pharmacology, molecular docking

## Abstract

Allergic rhinitis (AR) is one of the most prevalent allergic disorders worldwide. Current pharmacological treatments are often limited by suboptimal efficacy and notable adverse effects. Herbal medicines, with their multi-component and multi-target therapeutic characteristics, have attracted increasing attention. *Artemisia ordosica* Krasch. (AOK), a traditional Chinese/Mongolian medicine has demonstrated immunomodulatory, antioxidant, and anti-inflammatory activities. The anti-AR potential of AOK extract fractions was evaluated using in vitro mast cell degranulation inhibition assays, network pharmacology analysis, molecular docking, and molecular dynamics simulations to elucidate underlying pharmacological mechanisms. The P815 mast cell model induced by compound 48/80 was employed to assess the inhibitory activity and cytotoxicity of different extract fractions. Among the tested fractions, the ethyl acetate fraction exhibited the most potent inhibitory effect on mast cell degranulation without significant cytotoxicity. Network pharmacology analysis identified 254 potential AR-related targets of AOK, with Signal Transducer and Activator of Transcription 3(STAT3), Src protein(SRC), Tumor protein 53(TP53), AKT Serine/Threonine Kinase 1(AKT1), Heat Shock Protein 90 Alpha Family Class A Member 1(HSP90AA1), Estrogen Receptor 1(ESR1), and Phosphatidylinositol-4,5-bisphosphate 3-kinase catalytic subunit alpha(PIK3CA) identified as key hub proteins. Gene Ontology and KEGG pathway enrichment analyses indicated that AOK primarily modulated inflammatory and oxidative stress-related processes through the lipid and atherosclerosis, hypoxia-inducible factor-1, and AGE-RAGE signaling pathways. Molecular docking and dynamics simulations demonstrated strong binding affinities and stable interactions between major active constituents, particularly hydroxygenkwanin, and key targets such as SRC. The ethyl acetate fraction of AOK extract exhibited significant mast cell degranulation inhibitory activity, likely mediated via a synergistic multi-component, multi-target mechanism involving regulation of inflammatory and immune-related signaling pathways. These findings provide a pharmacological basis for the potential application of AOK in AR treatment.

## 1. Introduction

Associated with rapid industrialization, urbanization, and environmental changes, allergic rhinitis (AR) has become one of the most prevalent chronic respiratory inflammatory diseases worldwide, affecting approximately 10–40% of the global population. Recent epidemiological investigations have demonstrated that the prevalence of AR in China continues to rise without reaching a plateau, posing an increasingly serious public health burden [1,2]. AR is an immunoglobulin E (IgE)-mediated hypersensitivity disorder triggered by exposure to airborne allergens and characterized by T helper 2 (Th2)-driven mucosal inflammation [3]. Clinically, AR is characterized by nasal congestion, sneezing, rhinorrhea, nasal itching, and excessive nasal secretions. In contrast, severe or persistent cases are frequently associated with asthma, conjunctivitis, impaired sleep quality, and reduced quality of life [4,5].

At present, multiple therapeutic strategies are available for AR management, including allergen avoidance, pharmacotherapy, allergen-specific immunotherapy, and surgical intervention in selected patients [6]. However, although current first-line medications, including intranasal corticosteroids, antihistamines, and leukotriene receptor antagonists, are capable of alleviating certain clinical symptoms, relapse frequently occurs after treatment discontinuation. Moreover, long-term use is often associated with adverse effects, including nasal mucosal irritation, sedation, fatigue, and even hormonal disturbances [7,8]. More importantly, a considerable proportion of patients with moderate-to-severe AR fail to achieve satisfactory disease control despite receiving guideline-recommended therapies, which substantially impairs quality of life and work productivity [9]. Therefore, there remains an urgent clinical need to develop novel therapeutic agents that more effectively target the immunopathological pathways underlying AR while providing sustained symptom control and favorable long-term safety profiles, thereby overcoming the limitations of current treatment strategies.

*Artemisia ordosica* Krasch. (AOK), a traditional Chinese/Mongolian medicinal herb, has historically been described as possessing properties associated with resolving phlegm, clearing heat, and eliminating pathological fluids. According to the Chinese Materia Medica, AOK has traditionally been used to dispel wind-dampness, detoxify, and alleviate swelling, and has been applied in the treatment of conditions such as rheumatoid arthritis, common cold-related headaches, sore throat, and abscesses or boils [10]. The roots of AOK have been documented for the management of epistaxis in Medicinal Plants of China’s Desert Regions, with similar hemostatic applications also recorded in Inner Mongolia Chinese Herbal Medicine [11,12]. In addition, favorable therapeutic outcomes in the treatment of AR have been reported in traditional Mongolian medicine. Modern pharmacological studies have demonstrated that AOK is rich in flavonoids and terpenoids, which exhibit immunomodulatory, antioxidant, anti-inflammatory, and antibacterial activities [13]. However, the precise molecular mechanisms underlying its therapeutic effects against AR remain to be fully elucidated.

**Figure 1:**
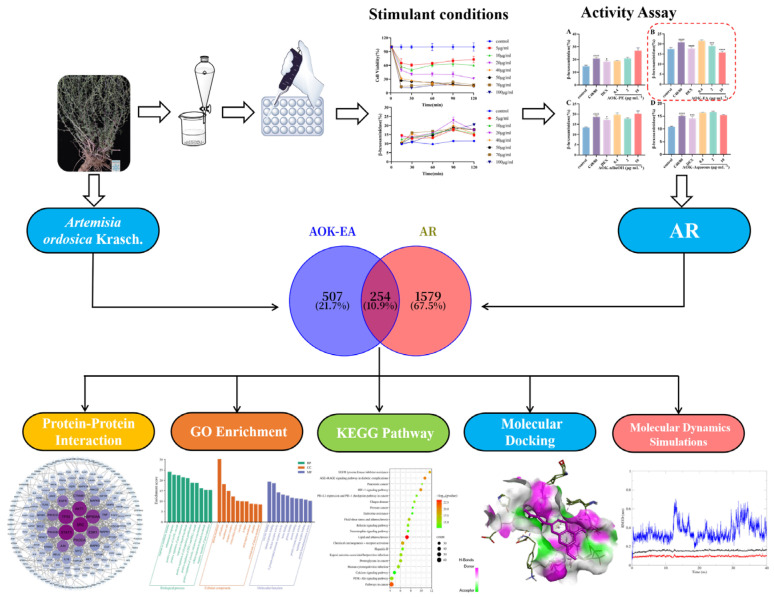
This is a schematic diagram that illustrates the overall workflow of this study. Note: The images of AOK are sourced from the Plant Photo Bank of China (PPBC).

In this study, an in vitro P815 mast cell degranulation inhibition model was established to screen the anti-AR activity of different fractions of AOK extract. Subsequently, network pharmacology approaches were applied to systematically predict the key bioactive constituents and potential molecular targets of the active fraction of AOK involved in the treatment of AR. Gene Ontology (GO) and Kyoto Encyclopedia of Genes and Genomes (KEGG) enrichment analyses were conducted to elucidate the biological processes and signaling pathways that may be regulated by these components. Finally, molecular docking and molecular dynamics simulations were performed to validate the stability of interactions between key compounds and core targets at the molecular level. This study will provide both theoretical support and experimental evidence for elucidating the pharmacodynamic basis and multi-target mechanisms underlying the therapeutic effects of AOK in the treatment of AR. The workflow diagram of this study is shown in Figure 1.

## 2. Results

### 2.1. Cell Growth Curve

**Figure 2:**
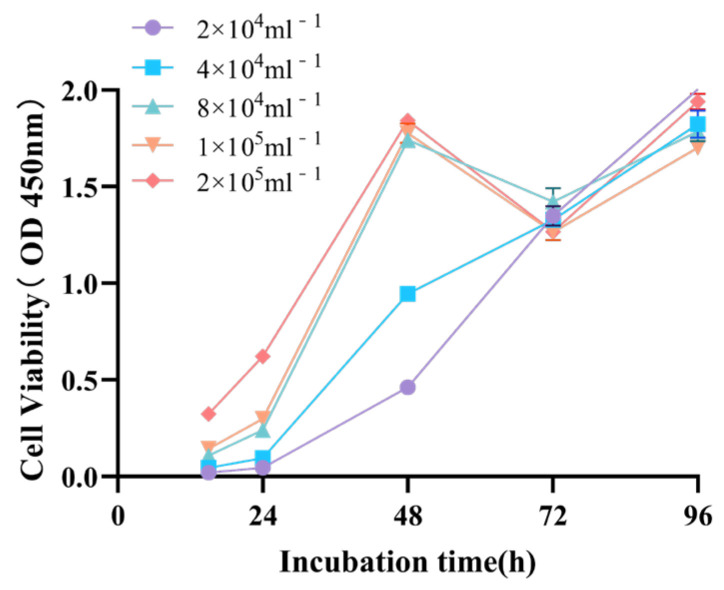
Growth curve of P815 cell lines.

Cell growth curves were generated using the CCK-8 assay to determine the optimal cell seeding density and experimental duration. As shown in Figure 2, P815 cells seeded at a density of 1.0 × 10^5^ cells/mL exhibited a more favorable and reproducible growth profile compared with other seeding densities. Under this condition, cell proliferation increased steadily and entered the logarithmic growth phase between 24 and 48 h. In contrast, cells seeded at lower densities displayed delayed proliferation patterns, whereas higher seeding densities led to premature growth plateauing, likely due to contact inhibition or nutrient limitation. Accordingly, the 24–48 h interval was selected as the optimal time window for subsequent in vitro experiments to ensure that the cells were maintained in an active proliferative state.

### 2.2. Effects of AOK Fractions on P815 Cell Viability

**Figure 3:**
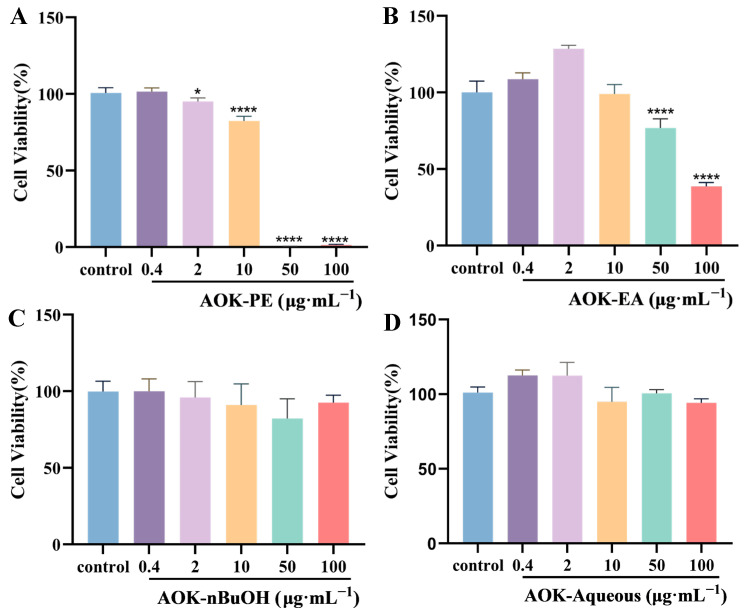
Effect of different extraction fractions of AOK on the survival rate of P815 cells (x ± s, n = 3). (**A**) PE fraction. (**B**) EA fraction. (**C**) *n*BuOH fraction. (**D**) Aqueous fraction. Each value represents the mean ± SD (* *p* < 0.05, **** *p* < 0.0001; unmarked *p* > 0.05 versus control group).

The effects of AOK PE, EA, *n*-BuOH, and aqueous extracts on P815 cell viability were evaluated using the CCK-8 assay. As shown in Figure 3, the results demonstrated that AOK-PE and AOK-EA reduced cell viability in a concentration-dependent manner. At low concentrations, minimal cytotoxicity was observed, whereas moderate to high concentrations (≥10 μg/mL) significantly decreased cell viability. Meanwhile, AOK-*n*-BuOH and AOK-aqueous did not induce significant changes in cell viability compared with the control group across the tested concentration range, indicating negligible cytotoxicity. These findings provided important evidence for the cellular safety evaluation of AOK and supported the subsequent screening of active components.

### 2.3. Establishment of the C48/80-Induced P815 Degranulation Model

**Figure 4:**
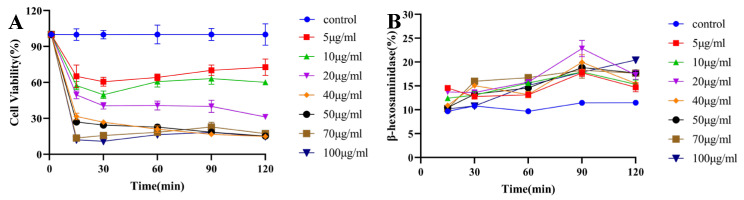
Exploring the modeling conditions of C48/80. (**A**) Effect of C48/80 on the survival rate of P815 cells. (**B**) Effect of C48/80 on the release of β-hexosaminidase from cells.

Compared with the control group, P815 cell viability decreased to below 50% when C48/80 concentrations exceeded 20 μg/mL, whereas prolonged exposure did not significantly affect cell viability (Figure 4A). C48/80-induced degranulation in P815 cells was assessed by measuring the β-hexosaminidase release rate. C48/80 treatment significantly increased the β-hexosaminidase release rate compared with the control group (*p* < 0.01). At a concentration of 20 μg/mL and a stimulation time of 1.5 h, the β-hexosaminidase release rate reached the highest level. The representative experimental results are presented in Figure 4.

### 2.4. Effects of AOK Fractions on P815 Cell Degranulation

**Figure 5:**
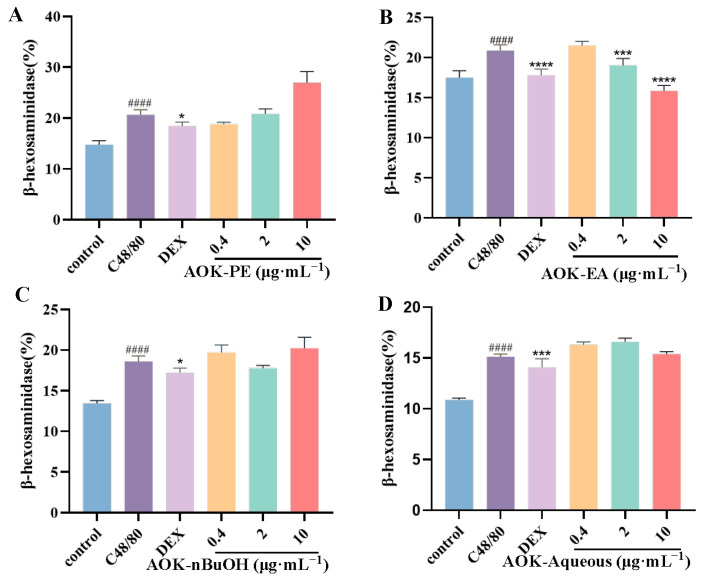
Effect of different extraction fractions of AOK on β-hexosaminidase release in P815 induced by C48/80 (x ± s, n = 3). (**A**) PE fraction. (**B**) EA fraction. (**C**) *n*BuOH fraction. (**D**) Aqueous fraction. Each value represents the mean ± SD. (* *p* < 0.05, *** *p* < 0.001, **** *p* < 0.0001, unmarked *p* > 0.05 versus C48/80 group, ^####^ *p* < 0.0001 versus control group).

The effects of different AOK extract fractions on P815 cell degranulation were evaluated using the β-hexosaminidase release rate as an indicator. Compared with the control group, C48/80 treatment significantly induced β-hexosaminidase release (*p* < 0.0001), indicating a pronounced degranulation response in P815 cells. The inhibitory effects of the various extract fractions on mast cell degranulation were further assessed, revealing notable differences among the fractions. Specifically, AOK-EA significantly reduced C48/80-induced β-hexosaminidase release (*p* < 0.0001), with a release rate reduced to approximately 76% of that in the C48/80 group. The positive control group exhibited a release rate of approximately 85% relative to the C48/80 group (*p* < 0.0001), whereas the other fractions showed no significant inhibitory activity. These results indicated that, within the tested concentration range (0.4–10 μg/mL), AOK-EA markedly suppressed mast cell degranulation, demonstrating potent inhibitory activity. Representative experimental results are presented in Figure 5.

### 2.5. Active Components of AOK and Their Potential Targets

**Figure 6:**
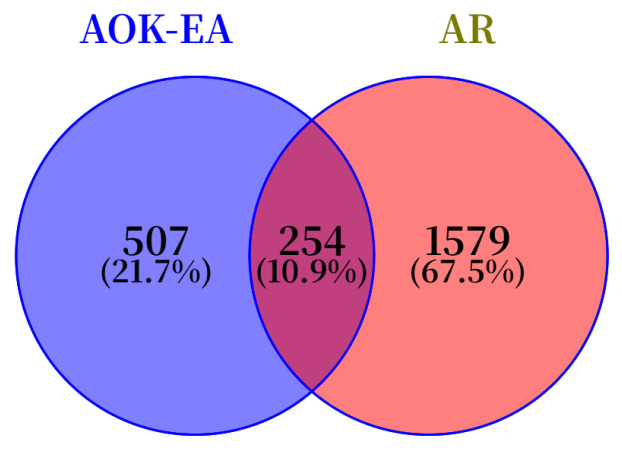
Venn diagram of AOK and AR targets.

A total of 3572 potential targets corresponding to these active components in the AOK-EA fraction were identified through integration of the TCMSP and SwissTargetPrediction databases. After removal of duplicate entries, 761 unique targets were retained (Figure 6).

### 2.6. AR Disease Targets

In total, 1786 targets related to AR were identified from the GeneCards database, whereas 3 and 446 targets were obtained from the OMIM and DisGeNET databases, respectively. After merging targets retrieved from these three disease-related databases and removing duplicate entries, 1833 unique potential targets associated with AR were retained (Figure 6).

### 2.7. Construction of the PPI Network

**Figure 7:**
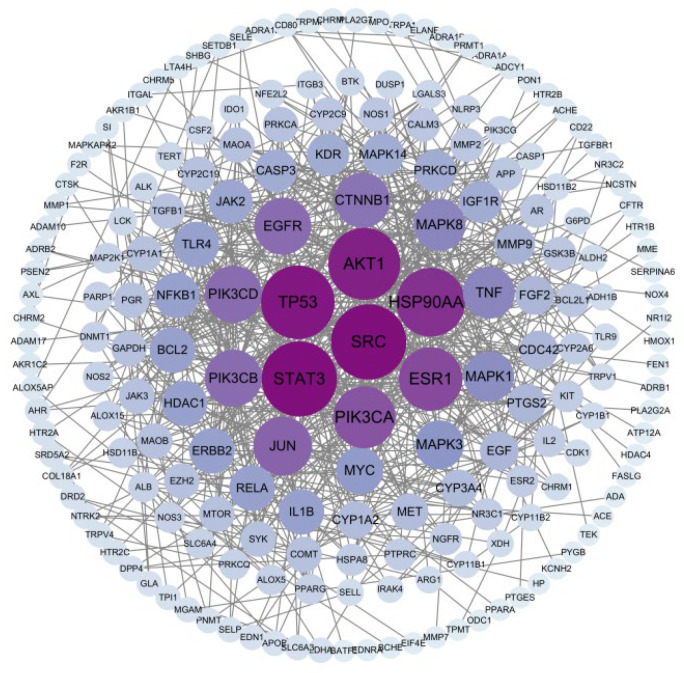
PPI network diagram of AOK in the treatment of AR.

**Figure 8:**
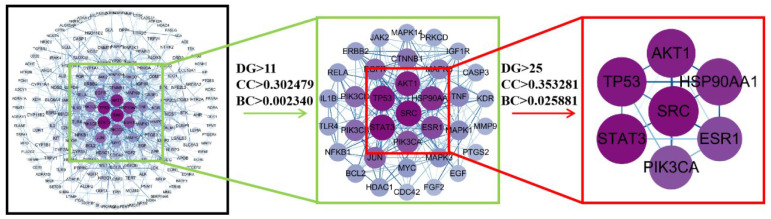
The filtering strategy diagram of key nodes. Note: DG stands for Connectivity; BC stands for intermediary centrality; CC stands for Proximity to Centrality.

Potential targets were analyzed for PPIs using the STRING database. The resulting data were imported into Cytoscape version 3.10.0 for topological analysis. A total of 191 nodes and 632 edges were identified in the constructed PPI network, indicating extensive interactions among the predicted therapeutic targets associated with AOK against allergic rhinitis (Figure 7). Node size and color intensity were positively correlated with degree values, with larger and darker nodes representing proteins with higher topological importance and stronger connectivity within the network. Topological analysis of the PPI network using Cytoscape 3.10.0 identified 30 core targets with the highest degree values (Supplementary Table S1). Further topological significance based on degree centrality (DG), closeness centrality (CC), and betweenness centrality (BC) identified several core targets, including SRC, AKT1, TP53, STAT3, HSP90AA1, ESR1, and PIK3CA, suggesting that these proteins may play critical roles in the anti-allergic mechanism of AOK-EA (Figure 8).

### 2.8. Construction of the Drug–Component–Disease–Target–Pathway Network Diagram

**Figure 9:**
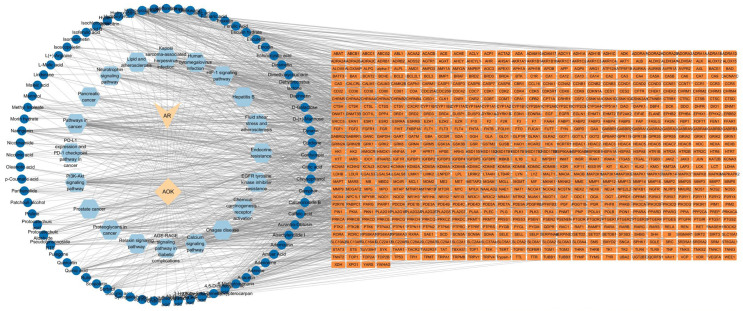
Drug–component–disease–target–pathway interaction network.

The potential active components of AOK-EA and the 254 shared component-disease targets were imported into Cytoscape. Components lacking intersections with targets were removed, and the top 20 pathways were simultaneously incorporated to generate a network diagram depicting the ‘drug–component–disease–target–pathway’ interactions (Figure 9). In this network, diamonds represent the herbal medicine, circles denote the active components in AOK-EA, rectangles indicate the 254 intersecting targets, hexagons correspond to the top 20 pathways, and V-shapes represent diseases. Based on the predefined topological parameters (DG > 25, CC > 0.353281, and BC > 0.025881), a total of seven targets, including STAT3, SRC, TP53, AKT1, HSP90AA1, ESR1, and PIK3CA, were identified as key targets within the network. Subsequently, network nodes were ranked according to their degree values. Nodes with a degree value greater than 39 were further selected as key active components, which included caffeic acid, hydroxycoumarin, methylnissolin, and hydroxygenkwanin.

Further pathway enrichment analysis based on *p*-values revealed that lipid and atherosclerosis, pathways in cancer, advanced glycation end products–receptor for advanced glycation end products (AGE-RAGE) signaling pathway in diabetic complications, hypoxia-inducible factor-1 (HIF-1) signaling pathway, and epidermal growth factor receptor (EGFR) tyrosine kinase inhibitor resistance were the top five significantly enriched pathways. Collectively, these core targets, bioactive compounds, and key signaling pathways might constitute a critical regulatory network through which the AOK-EA fraction exerts therapeutic effects against AR, thereby providing a promising natural product-based strategy for AR intervention. Each active compound was connected to multiple targets, and multiple targets were linked to various components. This network demonstrated that AOK-EA might exert therapeutic effects against AR through a multi-component and multi-target mechanism, acting via diverse pathways.

### 2.9. GO and KEGG Pathway Enrichment Analysis

**Figure 10:**
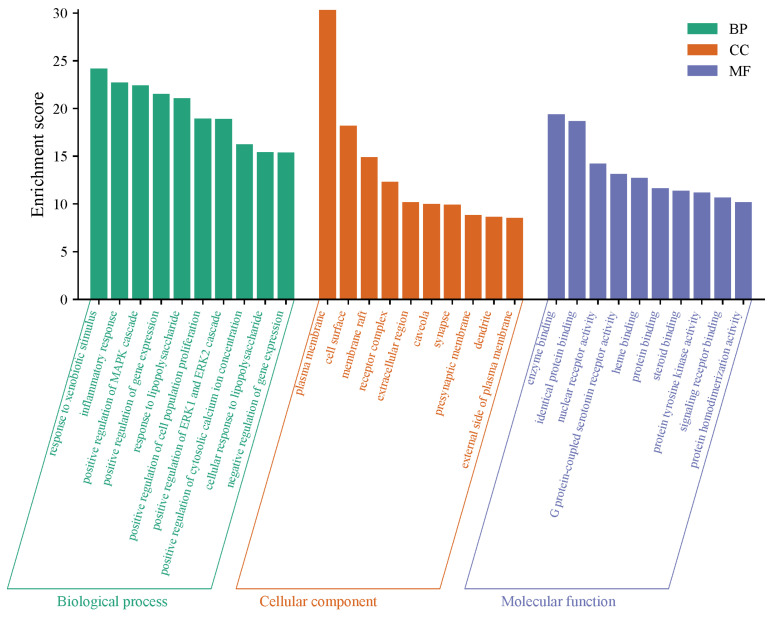
GO enrichment analysis bar chart.

GO analysis yielded 1136 entries, comprising 833 BP, 103 CC, and 200 MF. The top 10 entries for BP, CC, and MF, ranked according to *p*-values, were visualized as bar charts (Figure 10). BP terms were primarily associated with responses to external stimuli, inflammatory responses, and positive regulation of MAPK cascades. MF terms mainly included enzyme binding, identical protein binding, and nuclear receptor activity, while CC terms predominantly involved plasma membrane, cell surfaces, receptor complexes, and extracellular regions.

**Figure 11:**
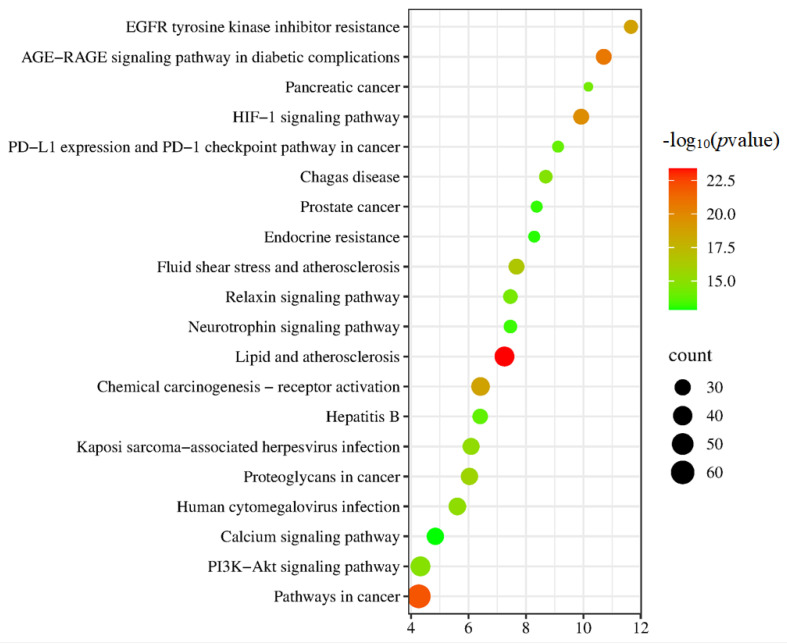
KEGG enrichment analysis bubble chart.

KEGG pathway enrichment analysis identified 183 pathways, with the top 20 pathways visualized according to ascending *p*-values (Figure 11). In the bubble plot, red bubbles represent lower *p*-values, whereas green bubbles indicate higher *p*-values; smaller *p*-values indicate greater significance, and larger bubble sizes correspond to a higher number of associated genes. Key signaling pathways implicated in AOK-EA-mediated treatment of AR included the lipid and atherosclerosis pathway, HIF-1 signaling pathway, calcium signaling pathway, and neurotrophin signaling pathway. Additionally, disease and cancer-related pathways, such as the advanced glycation end product-receptor for AGE-RAGE signaling pathway in diabetic complications and lipid and atherosclerosis-related pathways, were significantly enriched. These results suggested that the active components of AOK-EA might exert therapeutic effects against AR through multiple signaling pathways.

### 2.10. Molecular Docking

Molecular docking was performed between the top-ranked targets STAT3, SRC, TP53, AKT1, and HSP90AA1, with the key core components caffeic acid, hydroxycoumarin, methylnissolin, and hydroxygenkwanin to predict their binding potential. The detailed molecular docking binding energies are summarized in Table 1. Binding energy was inversely correlated with ligand–target affinity, with values below −5 kcal·mol^−1^ and −7 kcal·mol^−1^ generally indicating moderate and strong binding interactions, respectively [14]. Docking simulations conducted using AutoDock yielded the following binding energies with SRC: −9.63 kcal·mol^−1^ for caffeic acid, −8.29 kcal·mol^−1^ for hydroxycoumarin, −11.97 kcal·mol^−1^ for methylnissolin, and −12.87 kcal·mol^−1^ for hydroxygenkwanin. Among these interactions, hydroxygenkwanin exhibited the strongest binding affinity toward SRC.

**Figure 12:**
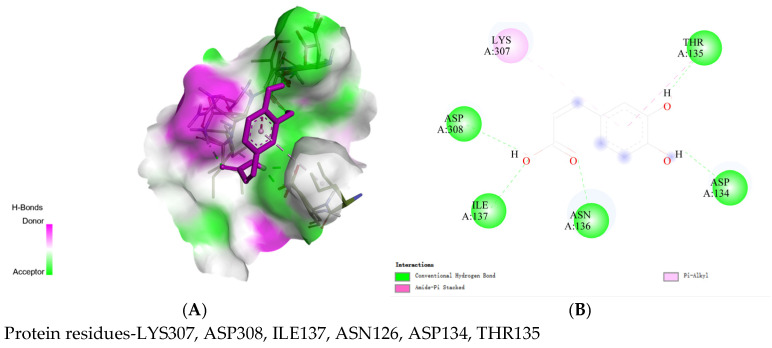

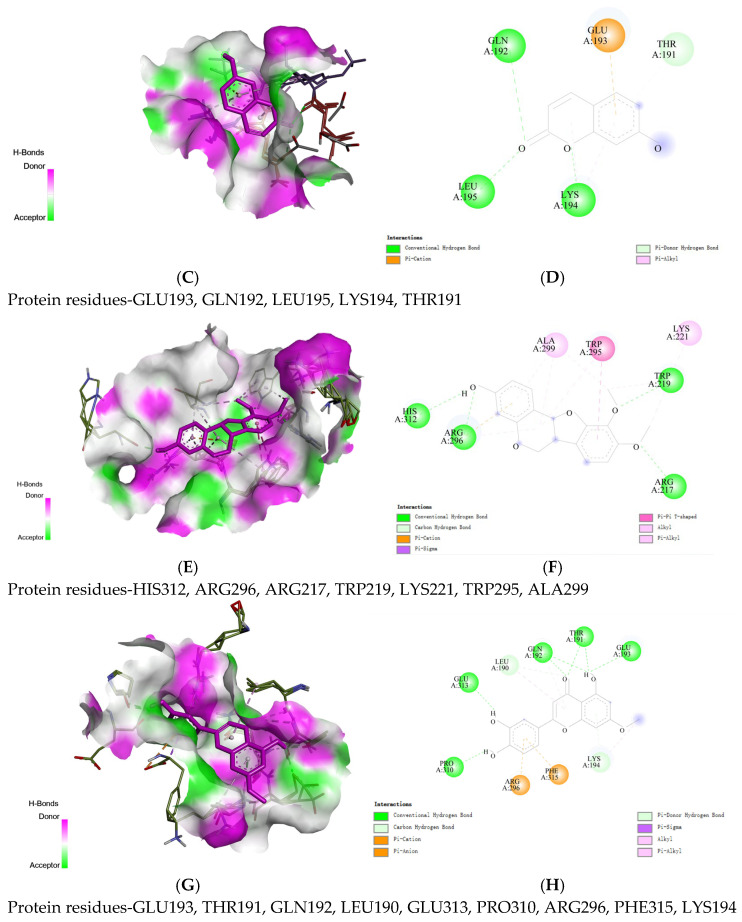
Molecular docking simulation of binding between the key components and core targets of AOK-EA. (**A**,**B**) 3D and 2D interaction structures of Caffeic acid with SRC, respectively. (**C**,**D**) 3D and 2D interaction structures of Hydroxycoumarin with SRC, respectively. (**E**,**F**) 3D and 2D interaction structures of Methylnissolin with SRC, respectively. (**G**,**H**) 3D and 2D interaction structures of Hydroxygenkwanin with SRC.

Molecular docking combined with two-dimensional interaction analysis demonstrated that all four key components could stably bind within the active pocket of SRC kinase. The binding modes of the key compounds with their corresponding targets are illustrated in Figure 12. Caffeic acid formed multiple conventional hydrogen bonds with residues ASP134, THR135, ASN136, and ASP308 via its hydroxyl and carboxyl groups, along with π-alkyl interactions with LYS307. This binding mode was predominantly governed by hydrogen bonding, complemented by hydrophobic interactions. Hydroxycoumarin primarily established hydrogen bonds with residues GLN192, LEU195, and LYS194, and displayed π-cation interactions with GLU193, indicating favorable polar interactions. Methylnissolin formed stable hydrogen bonds with HIS312 and ARG296, while its aromatic ring engaged in π-π stacking with TRP295 and hydrophobic interactions with ALA299 and LYS221, suggesting strong conformational complementarity. Hydroxygenkwanin interacted with SRC through multiple hydrogen bonds involving residues GLU313, GLN192, and THR191, as well as π-alkyl and π-cation interactions with PHE315 and ARG296, resulting in a more complex and stable binding network.

Overall, stable interactions between SRC and all four components were observed, mediated primarily by a combination of hydrogen bonding and hydrophobic interactions. Among these, the hydroxygenkwanin-SRC complex exhibited the strongest binding affinity, providing structural evidence supporting its potential biological relevance. These findings suggested that the identified compounds might play critical roles in mediating the anti-AR activity of AOK-EA.

### 2.11. Molecular Dynamics Simulation

**Figure 13:**
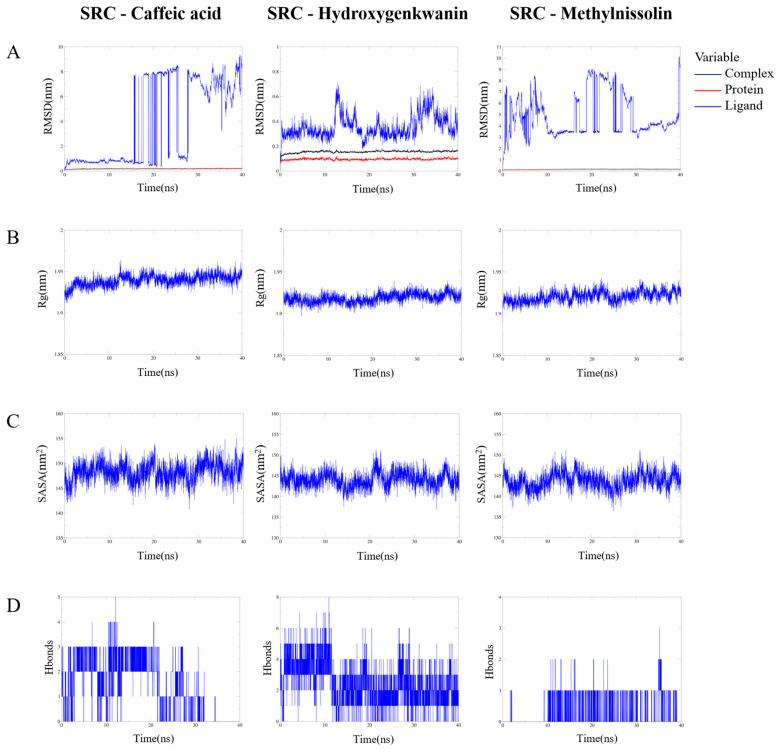
The results of the molecular dynamics simulation. (**A**) RMSD values of the three complexes. (**B**) Radius of gyration (Rg) values of the three complexes. (**C**) The evolution of the key distances between ligand features and the active site of the three complexes. (**D**) Number of hydrogen bonds in the three complexes.

To further investigate the stability of the protein–ligand interactions (with a binding energy threshold of ≤−9.63 kcal/mol), the top three docking complexes were selected for molecular dynamics (MD) simulations, including SRC–caffeic acid, SRC–methylnissolin, and SRC–hydroxygenkwanin, using GROMACS 2025.2. The MD simulation results, including RMSD, Rg, and SASA analyses (2025.2), provide an important basis for measuring the stability of protein–ligand complexes and the stability of protein tertiary structures after combining small molecules. As shown in Figure 13A, the backbone RMSD of SRC remained stable (~0.08–0.15 nm) across all systems, indicating that ligand binding did not significantly perturb the global protein conformation. Among the three complexes, hydroxygenkwanin exhibited the most stable binding behavior with minimal RMSD fluctuations, whereas caffeic acid and methylnissolin showed larger ligand RMSD variations, suggesting reduced binding stability and possible exploration of alternative binding modes. The Rg was analyzed to evaluate the overall compactness and conformational stability of the protein during the MD simulations. As shown in Figure 13B, the Rg values of SRC remained relatively stable within ~1.90–1.96 nm across all systems, indicating that ligand binding did not induce significant global conformational changes. Among the three complexes, the hydroxygenkwanin-SRC system exhibited the most stable Rg profile with minimal fluctuations, whereas the caffeic acid–SRC complex showed slightly higher Rg values and greater variability, suggesting minor reductions in structural compactness. Overall, these results indicate that the SRC protein maintained a stable and compact conformation throughout the simulation, with hydroxygenkwanin causing the least structural perturbation. SASA analysis showed that the values of SRC remained stable (~140–152 nm^2^) across all systems during the MD simulations, indicating that ligand binding did not cause significant structural expansion. Among the complexes, hydroxygenkwanin exhibited the lowest and most stable SASA values, whereas caffeic acid showed slightly higher fluctuations, suggesting increased solvent exposure due to ligand mobility. Overall, these results further support the greater stability of the hydroxygenkwanin-SRC complex (Figure 13C). The hydroxygenkwanin-SRC complex maintained a higher and more persistent number of H-bonds throughout the trajectory, indicating stable polar interactions within the binding pocket. In contrast, caffeic acid and methylnissolin exhibited fewer and more transient H-bonds, suggesting weaker and less stable binding interactions. These results further support the superior binding stability of hydroxygenkwanin with SRC (Figure 13D).

### 2.12. Overall Binding Free Energy Components (MM/PBSA)

**Figure 14:**
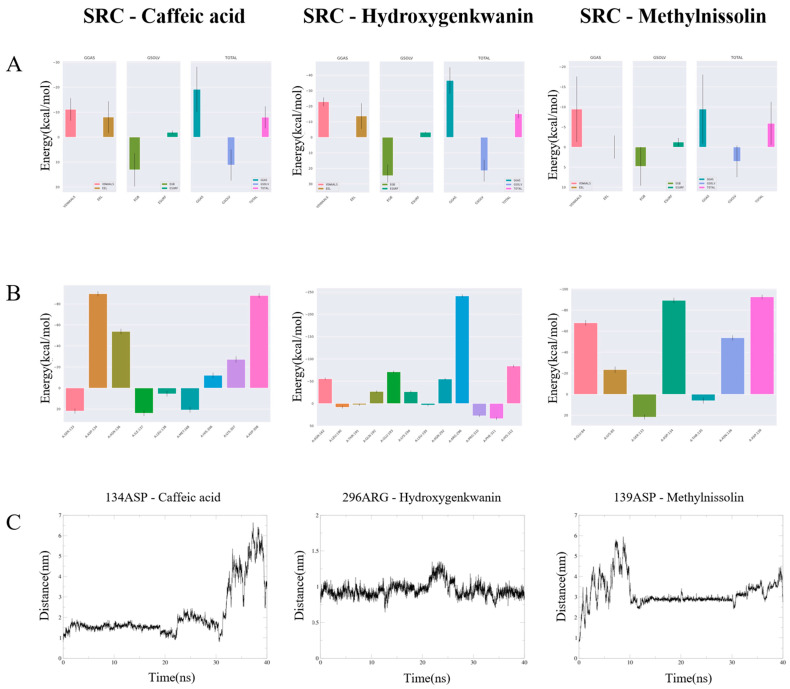
MM/PBSA Binding Free Energy Analysis and Residue-Level Interaction Dynamics of Key Ligand-Protein Complexes. (**A**) Overall binding free energy components for each ligand; (**B**) per-residue energy decomposition highlighting key contributing residues; (**C**) atomic-level contact dynamics showing residue–ligand distances over simulation time.

To quantitatively evaluate the binding affinities and identify the driving forces behind the molecular recognition, the total binding free energies (ΔG_bind) and their individual components were calculated via the MM/PBSA method (Figure 14A).

The hydroxygenkwanin-SRC complex exhibited the most favorable thermodynamic profile, with a total binding free energy of approximately −15 kcal/mol. This superiority is primarily driven by a dominant van der Waals contribution (ΔE ≈ −23 kcal/mol), significantly exceeding that of caffeic acid (−11 kcal/mol) and methylnissolin (−9 kcal/mol). Such high vdW values suggest optimized shape complementarity and tight hydrophobic packing between hydroxygenkwanin and the SRC binding pocket.

A key observation across all systems was that, while the gas-phase interactions (especially vdW and electrostatic interactions) provided the primary driving forces, the polar solvation energy (EGB) acted as the major energetic penalty. For hydroxygenkwanin, its superior gas-phase total (ΔG ≈ −37 kcal/mol) ultimately overcame the desolvation cost, resulting in the strongest overall binding affinity. Conversely, the high standard deviations observed for methylnissolin reflected substantial conformational variability, further validating the instability previously identified in the RMSD trajectories.

### 2.13. Per-Residue Energy Decomposition and Atomic-Level Contact Analysis

Per-residue energy decomposition using the MM/PBSA method was performed to elucidate the thermodynamic basis of ligand binding (Figure 14B). In the hydroxygenkwanin-SRC complex, Arg296 contributed most significantly to binding energy (≈ −200 kcal/mol), forming a persistent interaction confirmed by a stable distance of ~1.0 nm throughout the 40 ns simulation (Figure 14C). In contrast, key residues in the caffeic acid and methylnissolin complexes (Asp134 and Asp139) showed transient contributions, with distances fluctuating dramatically, reflecting weak or non-specific interactions. These results indicate that hydroxygenkwanin achieves superior binding stability through a strong and persistent interaction with SRC, supporting its potential as a promising inhibitor candidate.

## 3. Discussion

AR, referred to as “Bi Qiu” in the TCM system, has a long history of prevention and treatment in traditional clinical practice. With the advancement of TCM and increasing global research efforts, single herbal medicines, compound formulations, and bioactive constituents have demonstrated notable efficacy in alleviating clinical symptoms, delaying disease progression, and reducing recurrence rates in AR patients. As a traditional Chinese/Mongolian medicine, AOK is characterised by therapeutic properties described in TCM theory as dispelling wind-dampness, eliminating phlegm, and clearing heat. Clinically, it has been widely used in the management of inflammation-related disorders, such as chronic rhinitis, sinusitis, pharyngitis, and sore throat.

### 3.1. Previous Studies on AOK Pharmacological Effects

Previous studies have provided experimental evidence for the anti-inflammatory and immunomodulatory activities of AOK. It was reported that AOK extract significantly alleviated dextran sulfate sodium-induced colitis in mice by increasing colon length, enhancing antioxidant enzyme activity, inhibiting inflammatory cell infiltration, suppressing tumour necrosis factor-α (TNF-α) and interleukin-6 (IL-6) production, and reducing malondialdehyde levels [15]. In our previous study, we demonstrated that AOK extract significantly decreased the arthritis index, paw swelling, and spleen and thymus indices, while modulating serum inflammatory cytokine levels in the collagen-induced rheumatoid arthritic rat model [16]. In addition, we established an ovalbumin (OVA)-induced AR model in guinea pigs and found that the AOK extract markedly reduced inflammatory infiltration in the nasal mucosa and significantly decreased serum inflammatory mediators, including histamine, interferon-γ (IFN-γ), and IL-2 [17]. These studies demonstrated that AOK exerted substantial therapeutic effects across multiple inflammation-related disease models, highlighting its potential role in regulating immune homeostasis in the regulation of immune homeostasis. The present study further confirmed the anti-allergic cellular activity and potential molecular mechanisms.

### 3.2. Pharmacological Potential of the AOK-EA Fraction

The EA fraction, obtained using moderately polar ethyl acetate, was enriched with a diverse array of semi-polar compounds, including polyphenols, flavonoids, and certain esters, which are generally associated with extensive structural diversity and high biological activity. This observation was consistent with our previous studies, in which the EA fraction displayed superior anti-inflammatory and antimicrobial effects in both xylene-induced ear swelling in mice and OVA-induced AR in guinea pigs, markedly reducing serum inflammatory mediators and pathological changes in the nasal mucosa [17]. Collectively, these results indicated that the EA fraction of AOK root extracts represents a promising source for the discovery of active anti-AR compounds and further mechanistic investigations. The moderate polarity might prioritize the EA fraction for the enrichment of bioactive natural products with favorable solubility and pharmacological properties.

### 3.3. Targets Analysis

By constructing a PPI network and applying topological analysis algorithms within Cytoscape, several potential core targets, including SRC, STAT3, TP53, AKT1, and HSP90AA1, were identified. SRC family kinases function as upstream signaling initiators in mast cell degranulation [18]. Modulation of SRC family kinase activity has been shown to effectively inhibit mast cell degranulation and suppress the development of allergic diseases [19]. Signal transducer and activator of transcription (STAT) 3 is a transcription factor activated by cytokines including IFNs, IL-2, IL-6, IL-10, IL-22, and epidermal growth factor, and plays a critical role in regulating both innate and adaptive immune responses [20]. Recent evidence suggests that IL-37 alleviates allergic inflammatory signaling in AR by inhibiting STAT6 and STAT3 activation [21]. Tumor protein p53 inducible nuclear protein 1 (TP53INP1), a p53-interacting tumor suppressor protein with antioxidant properties, has also been implicated in AR pathogenesis. Elevated expression of miR-155–5p in AR has been reported to suppress TP53INP1 expression, thereby impairing the function of type 2 innate lymphoid cells and exacerbating allergic inflammation [22].

### 3.4. Analysis of Potential Active Components

Accumulating experimental data indicate that enhanced microvascular permeability to macromolecules is an early pathological feature of acute inflammation and allergic reactions. Mast cell-derived mediators, including histamine and serotonin, have been widely recognized as key contributors to increased vascular permeability in multiple species [23]. In this study, the four key active components in the AOK-EA fraction, caffeic acid, hydroxycoumarin, hydroxygenkwanin, and methylnissolin, were identified by ranking network nodes according to degree values using Cytoscape. Caffeic acid has been reported to significantly suppress compound 48/80-induced scratching behavior and vascular hyperpermeability, accompanied by a marked reduction in histamine levels in vivo [24]. In addition, 7-hydroxycoumarin has been shown to attenuate allergic airway inflammation by reducing inflammatory mediators such as IL-4 and IL-5 in bronchoalveolar lavage fluid, thereby modulating the Th1/Th2 immune balance and exerting therapeutic effects in asthma models [25].

### 3.5. Biological Enrichment Analysis

GO enrichment analysis indicated that the anti-AR effects of AOK were primarily mediated through modulation of responses to external stimuli and inflammatory processes. Furthermore, KEGG pathway enrichment analysis revealed that these active components were proposed to form a multi-target interaction network by concurrently regulating key signaling pathways, including HIF-1, phosphoinositide 3-kinase/protein kinase B (PI3K/Akt), and AGE-RAGE pathways. Collectively, these findings suggested that the AOK-EA fraction might exert a systematic regulatory effect on the inflammatory cascade underlying AR. Moreover, these findings not only elucidated the material basis of AOK’s anti-AR activity but also provided novel theoretical support for the synergistic pharmacological mechanisms of its active constituents. However, the precise molecular mechanisms require further experimental validation.

Limited studies have directly examined the immunomodulatory role of the AGE-RAGE signaling pathway. However, accumulating evidence suggests that neuroimmunoendocrine regulatory networks function synergistically to maintain systemic homeostasis [26]. The AGE-RAGE signaling pathway might indirectly regulate immune responses through modulation of neuroendocrine signaling, providing a theoretical basis for future investigations of the molecular mechanisms underlying the therapeutic effects of AOK in AR. Previous studies have consistently demonstrated that aberrant activation of the PI3K/Akt signaling pathway represents a core pathological mechanism in AR [27,28]. It was reported that silymarin significantly inhibited mast cell degranulation and calcium mobilization, while suppressing cytokine and chemokine release via the PLCγ and PI3K/Akt signaling pathways [29]. In addition, montelukast was demonstrated to alleviate airway inflammation in allergic asthma mice by reversing the expression of HIF-1α and prolyl hydroxylase domain-containing protein 2 at both the mRNA and protein levels [30].

### 3.6. Molecular Docking and Molecular Dynamics Simulation

Molecular docking was also employed to predict the interactions between core active components and key target proteins. Notably, the relatively high binding affinity of these core components for SRC suggested that it might serve as a critical mediator of their anti-AR effects. Recent studies have highlighted the key role of the respiratory epithelium in initiating type 2 immune responses to allergens, with IL-33 acting as a rapidly released alarmin. Epithelial IL-33 secretion depends on SRC activation via a dual oxidase 1-mediated oxidative mechanism and forms a feed-forward loop through ST2 signaling [31]. In addition, another SRC family kinase, p56lck, was identified as essential for downstream signaling of thymic stromal lymphopoietin, promoting type-2 immune responses and allergic manifestations via upregulation of p56lck and STAT6 [32]. These observations highlighted SRC as a potential key target for anti-AR interventions.

To further elucidate the interaction mechanism, molecular dynamics simulations were conducted on the top three ligand–target complexes ranked by docking scores. The simulations corroborated the docking results and demonstrated that hydroxygenkwanin formed a highly stable complex upon binding to SRC. Combined RMSD and RMSF analyses indicated that the complex maintained excellent structural stability and low local flexibility throughout the 40 ns simulation. Further analyses of hydrogen bonding and binding free energy provided a molecular-level explanation for this stability, revealing that complex formation is energetically favorable. Stable van der Waals interactions and persistent hydrogen bonds were found to play critical roles in maintaining both the binding conformation and overall stability of the SRC–hydroxygenkwanin complex. Collectively, these computational results provided robust theoretical support, from a molecular dynamics perspective, for the conclusion that hydroxygenkwanin exerted its pharmacological effects by targeting SRC.

## 4. Materials and Methods

P815 cells were purchased from Shanghai Fuheng Biotechnology Co., Ltd. (Shanghai, China). RPMI-1640 medium and fetal bovine serum (FBS) were obtained from Gibco (-Thermo Fisher Scientific, Waltham, MA, USA). Ninety-six-well and 24-well culture plates were purchased from Costar (Corning Inc., Corning, NY, USA). Compound 48/80 (C48/80) and dimethyl sulfoxide (DMSO) were obtained from Sigma-Aldrich (Merck, St. Louis, MO, USA). Dexamethasone (DEX) and p-nitrophenyl N-acetyl-β-D-glucosaminide (PNP-NAG) were purchased from Shanghai Yuanye Bio-Technology Co., Ltd. (Shanghai, China). Cell Counting Kit-8 (CCK-8) and Triton X-100 were obtained from Beyotime Biotechnology (Shanghai, China).

### 4.1. Preparation of AOK Extract and Fractions

Whole plants of AOK were collected in October 2024 from the Kangbashi District, Ordos, China. After taxonomic identification, a voucher specimen was deposited at the Ordos School of Clinical Medicine, Inner Mongolia Medical University. The plant material was dried at 20 °C, ground into powder, and stored at 4 °C until use. For the in vitro anti-AR activity assay, 125 g of dried, powdered AOK root was placed in a round-bottom flask and extracted with 1250 mL of 95% ethanol. Ultrasonic extraction was performed three times, each for 30 min. The resulting extract was filtered and concentrated under reduced pressure. Sequential liquid–liquid extraction was then conducted using petroleum ether (PE), ethyl acetate (EA), and n-butanol (*n*-BuOH) with a water-to-organic solvent ratio of 1:1, with each extraction repeated three times. Each fraction was subsequently filtered and concentrated under vacuum using a rotary evaporator. All powdered fractions were stored at 4 °C for further analyses. For cell-based experiments, each dried fraction was dissolved in dimethyl sulfoxide (DMSO) to prepare a 100 mg/mL stock solution. Before treatment, the stock solutions were diluted with complete culture medium to final concentrations of 0.4, 2, 10, 50, and 100 μg/mL. The final concentration of DMSO in the culture medium was maintained below 0.1% (*v*/*v*). A vehicle control group containing an equivalent concentration of DMSO corresponding to that used in the highest treatment group was included to exclude potential solvent-related effects on the experimental system. The reported concentrations of the AOK fractions were based on the total dry mass remaining after solvent evaporation rather than the quantified content of individual bioactive constituents.

### 4.2. Measurement of Cell Growth

P815 cells were cultured in RPMI-1640 medium supplemented with 10% fetal bovine serum (FBS) and 1% penicillin–streptomycin under humidified conditions at 37 °C in an atmosphere containing 5% CO_2_. Cell proliferation was assessed using the CCK-8 assay. P815 cells were seeded into 96-well plates at varying densities (2 × 10^3^, 4 × 10^3^, 8 × 10^3^, 1 × 10^4^, or 2 × 10^4^ cells per well) and incubated at 37 °C for different time periods (15, 24, 48, 72, or 96 h). Subsequently, 10 µL of CCK-8 solution was added to each well and incubated for 2 h. Absorbance was measured at 450 nm using a microplate reader (BioTek, Winooski, VT, USA).

### 4.3. Measurement of Cytotoxicity of the Stimulants

P815 cells in the logarithmic growth phase were seeded into 96-well plates at a density of 1.0 × 10^5^ cells/mL. Cells were treated with varying concentrations (0.4, 2, 10, 50, and 100 µg/mL) of the PE, EA, *n*-BuOH, and aqueous fractions of AOK for 24 h. Subsequently, 10 µL of CCK-8 solution was added to each well and incubated for 2 h, after which the optical density (OD) was measured at 450 nm.

### 4.4. Establishment of a C48/80-Induced P815 Cell Degranulation Model

The release of β-hexosaminidase was measured as previously described [33]. P815 cells were seeded in 24-well plates at a density of 1.0 × 10^5^ cells/mL. Cells were incubated with Tyrode’s solution containing varying concentrations of C48/80 (5, 10, 20, 40, 50, 70, and 100 µg/mL) for 15, 30, 60, 90, or 120 min. After incubation, the plates were placed on ice for 10 min, followed by centrifugation, and the supernatant was collected. The cell pellet was lysed with 0.1% Triton X-100 for 30 min, centrifuged, and the resulting supernatant was collected. Subsequently, 50 µL of 2 mM PNP-NAG was added to the supernatant and incubated at 37 °C for 60 min. The reaction was terminated by adding 150 µL of stop solution (0.1 M Na_2_CO_3_/NaHCO_3_ buffer), and the OD was measured at 405 nm. The β-hexosaminidase release rate, representing the degranulation of P815 cells under different treatment conditions, was calculated using the following formula:

$$\mathrm{Degranulation}\;\mathrm{rate}\;\left(\%\right)\;=\;\frac{\mathrm{OD}\;\mathrm{supernatant}}{\mathrm{OD}\;\mathrm{supernatant}+\mathrm{OD}\;\mathrm{lysate}}\;\times \;100$$

### 4.5. Measurement of Cytotoxicity of the Fractions

P815 cells in the logarithmic growth phase were seeded into 96-well plates at a density of 1.0 × 10^5^ cells/mL. Cells were treated with varying concentrations (0.4, 2, 10, 50, and 100 µg/mL) of the PE, EA, *n*BuOH, and aqueous fractions of AOK for 24 h. Subsequently, 10 µL of CCK-8 solution was added to each well and incubated for 2 h, after which the OD was measured at 450 nm.

### 4.6. Measurement of Degranulation Inhibitory Activity of the Fractions

P815 cells were seeded into 24-well plates at a density of 1.0 × 10^5^ cells/mL. Following a 24 h co-incubation with tested fractions, including a positive control group treated with DEX (1 μmol/L), the cells were stimulated with Tyrode’s solution containing 20 μg/mL C48/80 for 90 min. The cells were then placed on ice for 10 min, centrifuged, and the supernatants were collected for β-hexosaminidase activity determination, as described in Section 4.4.

### 4.7. Screening of Related Targets of AOK Active Components

The natural products present in the ethyl acetate fraction of AOK-EA were identified by our research group through previous liquid chromatography-mass spectrometry analyses [17]. Potential targets corresponding to these active components were retrieved using the Traditional Chinese Medicine Systems Pharmacology Database and Analysis Platform (TCMSP, https://tcmspw.com/tcmsp.php, accessed on 20 April 2026) [34] and the SwissTargetPrediction database (http://www.swisstargetprediction.ch/, accessed on 20 April 2026) [35], with a screening criterion of probability > 0. Target information was subsequently standardized using the UniProt database (https://www.uniprot.org/), and duplicate entries were removed to generate a consolidated list of putative targets.

### 4.8. Screening of AR Disease Targets

AR-related targets were retrieved by searching the GeneCards database (https://www.genecards.org/) [36], the Online Mendelian Inheritance in Man (OMIM) database (https://www.omim.org/) [37], and the DisGeNET database (https://disgenet.com/) [38]. using the keywords “allergic rhinitis” and “anaphylactic rhinitis”. Duplicate target genes were subsequently removed. The online tool Venny 2.1.0 was employed to generate a Venn diagram illustrating the overlap between the predicted targets of active components in the ethyl acetate fraction of AOK-EA and AR-associated targets, thereby identifying the intersecting targets.

### 4.9. Protein–Protein Interaction (PPI) Network Construction

The common targets of AOK-EA natural products and allergic rhinitis were imported into the STRING database (https://string-db.org/) [39]. The option ‘Multiple proteins’ was selected, the species was restricted to Homo sapiens, and a confidence score threshold of ≥0.9 was applied. The resulting PPI data were exported in TSV format and subsequently imported into Cytoscape version 3.10.0 (https://cytoscape.org/) [40] for topological analysis. Targets exhibiting degree centrality, closeness centrality, and betweenness centrality values above the network average were identified as key targets for further network pharmacology analysis.

### 4.10. GO Enrichment and KEGG Pathway Analysis

The intersecting target genes were imported into the Database for Annotation, Visualization, and Integrated Discovery (DAVID) for GO enrichment analysis, including biological process (BP), cellular component (CC), and molecular function (MF), as well as KEGG pathway analysis. The enrichment results were ranked in ascending order based on *p*-values, and the top 20 KEGG pathways were selected for further analysis. Visualization of the enrichment results was performed using the Microbioinformatics online platform (http://www.bioinformatics.com.cn/).

### 4.11. Construction of the Active Ingredient-Target-Disease-Pathway Network

Using Cytoscape version 3.10.0, an integrated network linking the active components of AOK with allergic rhinitis-associated targets and enriched pathways was constructed to generate a “component–target–disease–pathway” network diagram. This network was subsequently used to identify and analyze the key bioactive constituents of AOK-EA involved in the treatment of AR.

### 4.12. Molecular Docking

The core active compounds were retrieved from the TCMSP, and their three-dimensional structures were downloaded in MOL2 format. The crystal structures of the target proteins, including STAT3 (PDB ID: 6NJS), SRC (PDB ID: 2PF8), TP53 (PDB ID: 6VA5), AKT1 (PDB ID: 8UW9), and HSP90AA1 (PDB ID: 3WHA), were obtained from the Protein Data Bank (PDB, https://www.rcsb.org/, accessed on 20 April 2026).

Protein preparation was performed using AutoDockTools version 1.5.7 [41]. Before docking, water molecules and irrelevant co-crystallized ligands were removed from the protein structures. Polar hydrogen atoms and Kollman charges were subsequently added, and the processed protein structures were saved in PDBQT format. Ligand structures were prepared through hydrogen atom addition, energy minimization, and torsional bond assignment before conversion into PDBQT format.

Molecular docking was carried out using a semi-flexible protocol in AutoDock, in which the receptor proteins were treated as rigid, while the ligands were allowed conformational flexibility. The binding sites were selected according to the positions of the co-crystallized ligands and the reported active-site residues in the corresponding PDB structures. Grid boxes were centered on the active pockets of the target proteins, and the grid dimensions were adjusted to completely encompass the predicted binding regions and ensure sufficient conformational sampling of the ligands.

Docking simulations were performed using the Lamarckian Genetic Algorithm, with 10 independent docking runs conducted for each ligand–target pair. The docking conformation with the lowest binding energy and the most favorable interaction profile was selected for subsequent analysis. The molecular interactions between ligands and target proteins were further visualized and analyzed using Discovery Studio Visualizer.

### 4.13. Molecular Dynamics Simulations

To further evaluate the stability of the protein–ligand interactions, the top three docking complexes with binding energies ≤ −9.63 kcal/mol, including SRC–caffeic acid, SRC–methylnissolin, and SRC–hydroxygenkwanin, were selected for molecular dynamics (MD) simulations using the GROMACS 2024.1 software package [42]. Protein topology files were generated using the pdb2gmx module with the CHARMM36 force field. The N- and C-termini of the proteins were assigned as NH3+ and COO− groups, respectively. Ligand topology and parameter files were generated using the online CHARMM General Force Field (CGenFF) server (https://app.cgenff.com/, accessed on 20 April 2026).

Each protein–ligand complex was placed in a cubic simulation box with a minimum distance of 1.0 nm from the box edge and solvated using the TIP3P water model. Appropriate numbers of Na+ and Cl− ions were added to neutralize the system and maintain ionic balance.

Energy minimization was subsequently performed using the steepest descent algorithm until the maximum force on any atom was less than 1000 kJ/mol/nm, thereby eliminating unfavorable steric contacts and ensuring system stability. Following minimization, equilibration was conducted in two consecutive phases, including a 500 ps NVT ensemble equilibration and a 1 ns NPT ensemble equilibration, both under position restraints applied to the heavy atoms of the protein.

During equilibration and production simulations, the temperature was maintained at 310 K using the V-rescale thermostat with a coupling constant of 0.1 ps, whereas the pressure was maintained at 1 bar using the Parrinello–Rahman barostat with a coupling constant of 2.0 ps. The integration time step was set to 2 fs. Short-range van der Waals and electrostatic interactions were calculated using a cutoff distance of 1.2 nm, while long-range electrostatic interactions were treated using the Particle Mesh Ewald (PME) method. Periodic boundary conditions were applied throughout the simulations.

Production MD simulations were performed for 40 ns for each system, and trajectory coordinates were recorded every 2 ps. To ensure reproducibility, three independent replicate simulations were conducted for each protein–ligand complex, and all trajectories were included in the subsequent analyses.

Trajectory analyses were performed using the built-in GROMACS utilities, including root mean square deviation (RMSD), root mean square fluctuation (RMSF), radius of gyration (Rg), solvent-accessible surface area (SASA), and hydrogen bond (H-bond) analyses, to comprehensively evaluate the conformational stability and dynamic properties of the protein–ligand complexes. The resulting data were visualized using QtGrace v0.2.6.

### 4.14. MM/PBSA Binding Free Energy Calculations

The binding free energies (ΔG_bind) of the protein–ligand complexes and their individual energy contributions were calculated using the g_mmpbsa tool based on the MD simulation trajectories. Snapshots were extracted every 100 ps from the final 20 ns of each equilibrated trajectory, resulting in a total of 200 frames per simulation for MM/PBSA analysis.

The polar solvation energy was calculated using the Poisson–Boltzmann model, whereas the nonpolar solvation energy was estimated based on the SASA. The TIP3P water model was applied throughout the calculations. The dielectric constants for the solvent and solute were set to 80 and 2, respectively.

In addition, per-residue free energy decomposition analysis was performed to identify the key amino acid residues contributing to ligand binding and complex stabilization.

### 4.15. Statistical Analysis

Statistical analyses were performed using GraphPad Prism 9.0 software. Between-group differences were evaluated by one-way analysis of variance (ANOVA) followed by multiple comparison tests. Data are presented as mean ± standard deviation (SD). In the figures, asterisks indicate statistical significance compared with the C48/80 group (* *p* < 0.05, ** *p* < 0.01, *** *p* < 0.001, **** *p* < 0.0001), and number signs (#) indicate statistical significance compared with the control group (^#^ *p* < 0.05, ^##^ *p* < 0.01, ^###^ *p* < 0.001, ^####^ *p* < 0.0001). Unmarked comparisons versus the C48/80 group were considered statistically non-significant (*p* > 0.05).

## 5. Conclusions

In summary, the EA fraction of AOK extract exhibited significant inhibitory effects on P815 mast cell degranulation, demonstrating potent anti-allergic activity. This effect is likely mediated by multiple bioactive constituents, including caffeic acid, hydroxycoumarin, methylnissolin, and notably hydroxygenkwanin. Among these compounds, hydroxygenkwanin demonstrated favorable binding characteristics toward SRC in network pharmacology analysis, molecular docking, molecular dynamics simulations, and MM/PBSA binding free energy calculations, suggesting that it may represent a potential bioactive constituent involved in the anti-allergic effects of AOK. These compounds interact with key signaling targets such as SRC, STAT3, and TP53, suggesting a multi-target mechanism of action. Network pharmacology and molecular simulations further indicated that several signaling pathways, including PI3K/Akt and HIF-1, may participate in the synergistic regulation of inflammatory responses. Collectively, the present findings provide evidence supporting the anti-allergic potential of the AOK-EA fraction and suggest that hydroxygenkwanin may serve as a promising candidate compound for further pharmacological investigation. Furthermore, this study provides a scientific basis for the pharmacological development of this traditional Chinese/Mongolian medicinal herb in the treatment of allergic rhinitis.

Nevertheless, several limitations of the present study should be acknowledged. First, the anti-allergic effects of AOK were primarily evaluated using in vitro mast cell models combined with computational analyses, which may not fully reflect the complex pathological processes of allergic rhinitis in vivo. Second, the chemical constituents used for the network pharmacology analysis were derived from compounds previously identified by LC-MS in earlier studies conducted by our research group. The specific chemical composition and the exact contents of the key compounds identified through network pharmacology screening were not quantitatively characterized in the current extract. Although the AOK materials used in the present study were not from the same batch as those reported previously, the different fractions investigated in this work were prepared using the same fractionation procedures. As a result, the specific chemical composition and the exact contents of the key compounds in the present AOK-EA fraction were not quantitatively characterized. In addition, although several potential active compounds were identified, their individual pharmacological contributions and possible synergistic effects have not yet been experimentally validated. Furthermore, the predicted target–pathway relationships were mainly based on bioinformatics analyses and molecular simulation approaches and therefore still require further biological verification through additional in vitro and in vivo experiments.

Future studies will focus on comprehensive LC-MS characterization of the EA fraction used in the present study to identify and quantify the key bioactive constituents. In particular, hydroxygenkwanin will be further investigated through mast cell degranulation assays, target-binding validation experiments, and mechanistic studies involving SRC–related signaling pathways. Additional in vivo experiments and molecular biology approaches, including gene silencing, overexpression, and pathway inhibition studies, will also be conducted to further elucidate the pharmacological mechanisms underlying the anti-allergic effects of AOK.

## Figures and Tables

**Table 1:** Docking binding energies of target molecules for active ingredients and their combined action.

S. Nos	Active Ingredient	Binding Energy (kcal/mol)				
		STAT3 (6NJS)	SRC (2PF8)	TP53 (6VA5)	AKT1 (8UW9)	HSP90AA1 (3WHA)
1	Caffeic acid	−4.33	−9.63	−6.28	−5.21	−4.66
2	Hydroxycoumarin	−4.52	−8.29	−6.36	−5.51	−5.36
3	Methylnissolin	−6.16	−11.97	−6.68	−6.15	−6.34
4	Hydroxygenkwanin	−5.63	−12.87	−6.67	−6.54	−6.37

## Data Availability

All the data generated or analyzed during the current study are included in the manuscript.

## Supplementary Materials

The following supporting information can be downloaded at: https://www.mdpi.com/article/10.3390/ijms27115122/s1.
