# Supplementary material for: Study of Artemisia ordosica Krasch. Against Allergic Rhinitis Based on the P815 Mast Cell Degranulation Model, Network Pharmacology, Molecular Docking, and Molecular Dynamics
Source: Int J Mol Sci. 2026 Jun 5;27(11):5122. doi: 10.3390/ijms27115122 (PMC13257801; doi:10.3390/ijms27115122)
Supplement: Supplementary file 1 [file ijms-27-05122-s001.zip › ijms-4299513-supplementary.pdf]

Table S1. The core target of AOK in the treatment of AR.

S. nos	Target	Degree	Betweenness	Closeness
1	STAT3	33	0.119483	0.383648
2	SRC	33	0.111445	0.356725
3	TP53	32	0.082548	0.372709
4	AKT1	31	0.084383	0.368952
5	HSP90AA1	29	0.105440	0.372709
6	ESR1	26	0.031709	0.354651
7	PIK3CA	25	0.025882	0.353282
8	JUN	23	0.040583	0.344633
9	EGFR	22	0.039515	0.358824
10	PIK3CB	22	0.016958	0.332727
11	PIK3CD	22	0.016958	0.332727
12	CTNNB1	21	0.013317	0.341418
13	MAPK8	19	0.014241	0.337017
14	TNF	19	0.082468	0.366000
15	MAPK1	18	0.020727	0.353965
16	MAPK3	17	0.020087	0.353282
17	MYC	16	0.004686	0.325623
18	RELA	15	0.006773	0.321053
19	ERBB2	15	0.002340	0.326786
20	NFKB1	15	0.006382	0.321053
21	TLR4	15	0.030155	0.326786
22	IL1B	15	0.033319	0.334552
23	HDAC1	15	0.013046	0.313894
24	BCL2	15	0.017744	0.336397
25	JAK2	14	0.005591	0.313894
26	IGF1R	13	0.006117	0.320490
27	KDR	13	0.012765	0.314433
28	CASP3	13	0.066128	0.345936
29	MAPK14	13	0.003565	0.309645
30	PRKCD	13	0.018725	0.317158
